# Variability in pharmacologically-induced coma for treatment of refractory status epilepticus

**DOI:** 10.1371/journal.pone.0205789

**Published:** 2018-10-31

**Authors:** Jingzhi An, Durga Jonnalagadda, Valdery Moura, Patrick L. Purdon, Emery N. Brown, M. Brandon Westover

**Affiliations:** 1 Massachusetts Institute of Technology, Cambridge, Massachusetts, United States of America; 2 Harvard-MIT Division of Health Science and Technology, Cambridge, Massachusetts, United States of America; 3 Massachusetts General Hospital, Harvard Medical School, Cambridge, Massachusetts, United States of America; 4 MIT Department of Brain and Cognitive Sciences, Cambridge, Massachusetts, United States of America; 5 Institute of Medical Engineering and Sciences, Cambridge, Massachusetts, United States of America; University of Toronto, CANADA

## Abstract

**Objective:**

To characterize the amount of EEG suppression achieved in refractory status epilepticus (RSE) patients treated with pharmacologically-induced coma (PIC).

**Methods:**

We analyzed EEG recordings from 35 RSE patients between 21–84 years-old who received PIC that target burst suppression and quantified the amount of EEG suppression using the burst suppression probability (BSP). Then we measured the variability of BSPs with respect to a reference level of BSP 0.8 ± 0.15. Finally, we also measured the variability of BSPs with respect to the amount of intravenous anesthetic drugs (IVADs) received by the patients.

**Results:**

Patients remained in the reference BSP range for only 8% (median, interquartile range IQR [0, 29] %) of the total time under treatment. The median time with BSP below the reference range was 84% (IQR [37, 100] %). BSPs in some patients drifted significantly over time despite constant infusion rates of IVADs. Similar weight-normalized infusion rates of IVADs in different patients nearly always resulted in distinct BSPs (probability 0.93 (IQR [0.82, 1.0]).

**Conclusion:**

This study quantitatively identified high variability in the amount of EEG suppression achieved in clinical practice when treating RSE patients. While some of this variability may arise from clinicians purposefully deviating from clinical practice guidelines, our results show that the high variability also arises in part from significant inter- and intra- individual pharmacokinetic/pharmacodynamic variation. Our results indicate that the delicate balance between maintaining sufficient EEG suppression in RSE patients and minimizing IVAD exposure in clinical practice is challenging to achieve. This may affect patient outcomes and confound studies seeking to determine an optimal amount of EEG suppression for treatment of RSE. Therefore, our analysis points to the need for developing an alternative paradigm, such as vigilant anesthetic management as happens in operating rooms, or closed-loop anesthesia delivery, for investigating and providing induced-coma therapy to RSE patients.

## Introduction

Refractory status epilepticus (RSE) is a life threatening condition with a mortality rate of up to 40%. [1, 2] It is defined by generalized or focal continuous seizures that fail to respond to first and second line pharmacological treatment. [3] International guidelines advocate treating RSE with pharmacologically-induced coma achieved with a continuous infusion of intravenous anesthetic drugs (IVADs), such as midazolam, propofol, and/or barbiturates. This treatment aims to suppress brain activity in order for normal physiology to resume and abort seizures. [3–5] It requires a careful balance between maintaining sufficient brain inactivation and minimizing the risks of IVAD exposure. [3,4] As brain inactivation cannot be measured easily, clinicians typically use a distinctive pattern on the electroencephalogram (EEG) called burst suppression as a surrogate to guide titration of IVADs. The burst suppression pattern consists of alternating periods of high (‘bursts’) and low (‘suppressions’) voltage. Although anesthetics may have differential effects on excitatory and inhibitory neuronal activity [6,7] the overall effect of burst suppression is a profound global reduction in overall neuronal activity, including seizure activity. [8]

Delivering PIC therapy for RSE patients is challenging because it requires frequent patient monitoring, subjective interpretation of the EEG, and manual titration of IVADs by busy intensive care staff for prolonged periods often lasting 24–48 hours. [9] Moreover, subspecialists who are trained to interpret EEG and administer anesthetic drugs are often unavailable, leaving the tasks to non-experts. As a result, many have questioned the quality of induced-coma provided to RSE patients and have searched for ways to improve the therapy. [10,11]

In this investigation, we examined the challenges associated with the delivery of PIC in RSE patients by providing a quantitative assessment of the amount of EEG suppression achieved under current practice.

## Materials and methods

### Clinical data collection

We collected archived clinical data and EEGs from 35 consecutive patients who received PIC that targeted burst suppression for treatment of RSE in the neurological intensive care unit of Massachusetts General Hospital (MGH) between May 2012 and November 2014. This study was approved by the MGH Human Subjects Research Committee. All patient data were fully anonymized before analysis and patient consent was not required. We analyzed data from patients with anoxic etiologies of RSE (aRSE) separately from patients with non-anoxic etiologies of RSE (nRSE). All EEGs were recorded using nineteen silver/silver chloride electrodes affixed to the scalp according to the international 10–20 system. Data were recorded at 256 or 512 Hz, using XLTEK clinical EEG equipment (Natus Medical Inc., Oakville, Canada). Clinical information including time-stamped doses of all administered medications and vital signs were extracted from hand-written and electronic medical records.

### EEG analysis and quantification of burst suppression

We preprocessed EEGs with an independent component analysis (ICA)-based artifact reduction algorithm (Persyst 12, San Diego, CA). Artifact-reduced EEGs were exported in European Data Format, then converted into MATLAB format (Natick, MA). Further artifact removal was done by rejecting epochs of data with high amplitude (>500uV).^17^ Validity of the artifact-reduction was confirmed by visual inspection. Then, artifact-reduced EEGs were band-pass filtered at 0.5–55 Hz and mapped to an average montage. Next, we used a previously validated algorithm to convert the preprocessed EEGs into binary signals with zeros presenting bursts and ones representing suppressions. [12] Applying this algorithm to each channel yielded nineteen single-channel binary signals for each patient. Then, we obtained a global binary signal that integrates information across all channels using a published voting algorithm. [13] The global binary signal is more noise resilient than the binary signal computed from individual channels. [13] Finally, we calculated burst suppression probabilities (BSPs) from the global binary signals. BSP represents the instantaneous probability that an EEG epoch is in the suppressed state. [14] BSP is similar to the more traditional burst suppression ratio (BSR) at steady state, but is better for dynamical data, and has the advantage that it is a well-defined probability that allows for formal statistical comparison of the level of burst suppression across time. [15] Nevertheless, on the minutes-to-hours timescale that is relevant to this study, the difference between BSR and BSP is minimal.

### Measuring the amount of EEG suppression achieved in each patient

We summarized the amount of EEG suppression achieved as the extent to which the measured BSPs agreed with reference BSPs. The reference BSPs are inferred from the institutional (MGH) management guideline for RSE, which advises a goal of one burst per ten seconds, or 80% suppressions. [16] Because bursts typically last one to two seconds, this requirement was conservatively interpreted as equivalent to BSPs of 0.8 ± 0.15. [11,12] We compared measured BSPs with reference BSPs only for periods when there is documented intent in the medical record to maintain the studied patients in burst suppression. Specifically, we computed the percentage of time each patient spent above the reference range (PTa, BSP >0.95), within the reference range (PTi, 0.65≤ BSP ≤0.95) and below the reference range (PTb, BSP <0.65).

### Comparison of the amount of EEG suppression achieved in patient groups

We used probabilities that a patient group is accurately (and reliably) controlled, p_a_ (and p_r_), to describe and compare the quality of burst suppression achieved in the nRSE and aRSE groups. To compute these probabilities, we first defined for each patient a binary status of either accurately (reliably) or inaccurately (unreliably) controlled. Control is *accurate* when the measured BSPs were statistically indistinguishable from BSP 0.8 with 95% confidence. Control is *reliable* when the absolute difference between the measured BSPs and BSP 0.8 was less than 0.15 with 95% confidence. The status of accurate (reliable) control for each patient is treated as a binary observation sampled from a binomial model for accurate (reliable) control parameterized by p_a_ (p_r_). Given the observations and assumption of a uniform prior, we find a posterior probability distribution for different possible values of p_a_ (p_r_), and draw 100,000 Monte Carlo samples of p_a_ (p_r_) from the distribution [11]. nRSE and aRSE groups have different parameters denoted as p_a,nRSE_ (p_r,nRSE_) and p_a,aRSE_ (p_r,aRSE_). The probability that the aRSE group was more accurately (reliably) controlled than the nRSE group is the fraction of draws for which the Monte Carlo samples of p_a,aRSE_ (p_r,aRSE_) were larger than the Monte Carlo samples of p_a,nRSE_ (p_r,nRSE_).

### Amount of EEG suppression during constant rate(s) of IVAD infusion(s)

We identified constant-dose data segments when patients received constant rates of IVAD infusion(s) for at least 2 hours, and analyzed the measured BSPs during these periods. We excluded data 30 minutes before and after any recorded change in rates of IVAD infusion(s) and discarded constant-dose data segments affected by boluses. We fitted linear regression to capture the drift of the measured BSPs over time within each constant-dose data-segments.

## Results

**Fig 1:**
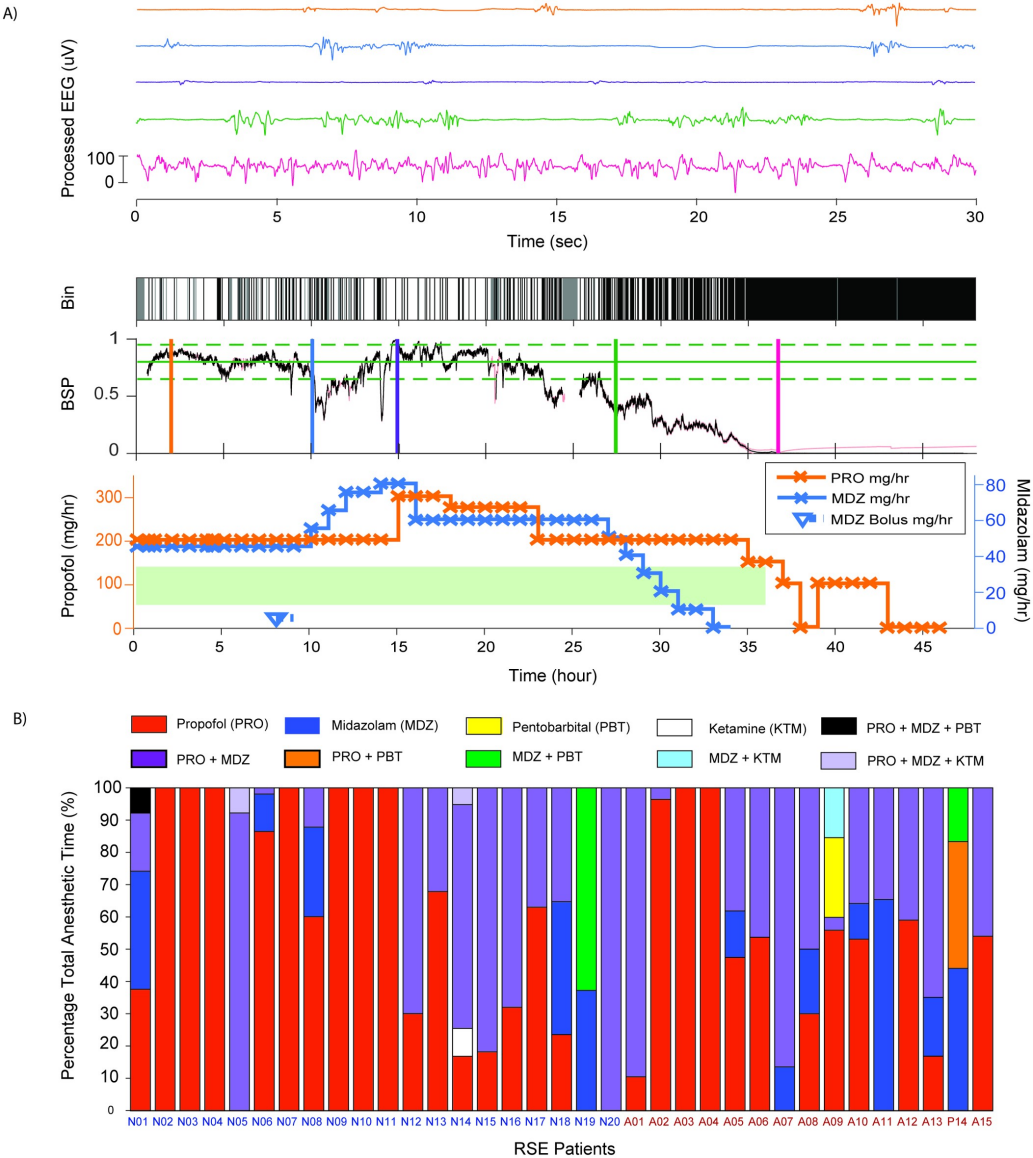
Visual summary of data. A) Example of EEG processing and clinical data collected for one patient. This panel illustrates the correlation made between the EEG and clinical data gathered. (Top) 30-second segments of EEG at selected points of time to illustrate the relationship between the BSP values and the time domain EEG pattern. (Bottom) 48 hours of binary EEG (Bin), BSP and medication used. In the binary EEG plot, suppressions are white and non-suppression periods are black. In the plot of BSP, the target BSP of 0.8 is indicated by the solid green line; the upper and lower bound of the reference range (i.e. BSP 0.65 and BSP 0.95) are indicated by the dashed green line; and the measured BSPs are shown in black. In the medication plot, solid lines show the rates of infusion of IVADs used and hollow markers indicate boluses. Periods with intent to achieve EEG burst suppression are highlighted with a light green bar. B) Overview of all patient profiles and infusion medication used. Percentage of total anesthetic time for each medication or combinations of medications used. Refer to the legend of Table 1 for annotations of abbreviations.

Thirty-five patients who underwent PIC for treatment of RSE were included in the study. We assigned fifteen patients with RSE that was preceded by a period of anoxia to the aRSE group (labeled A01 –A15), and the remaining twenty patients to the nRSE group (labeled N01 –N20). Data collected for each patient are summarized in (Fig 1a and Table 1). A total of 2944 hours of drug-dose-matched EEG data were available for analysis across all 35 patients. In order to assess the levels of brain inactivation achieved, as analyzed 1954 hours (66%) of the periods where there was documented intent to keep patients in burst suppression.

**Table 1:** Summary characteristics of patients and etiology.

	No. of Patients	Age Range (median)	Gender	Medical Diagnosis	Seizure Type (no)	IVAD and Dex
nRSE	20	21–75 (58)	12F 8M	Encephalitis (3) TBI (3) Tumor (3) Epilepsy (3) Drug / Metabolic (3) Stroke (1) NORSE (4)	NCSE (7) GCSE(8) EPC(1) Focal Motor(4)	MDZ (13) PRO(20) KTM (3) Dex (1)
aRSE	15	26–84 (60)	4F 11M	Anoxic Brain Injury (all) Cardiac arrest (14) Respiratory failure and shock (1)	NCSE (2) Focal Motor (1) MSE (12)	MDZ (13) PRO(14) PTB (2)

Legend to abbreviations: nRSE—non-post-anoxic epilepticus patients, aRSE—anoxic epilepticus patients, TBI—traumatic brain injury, NORSE—new onset refractory status epilepticus of unknown origin, NCSE—non-convulsive status epilepticus, GCSE—generalized convulsive status epilepticus, EPC—epilepsia partialis continua, MSE—myoclonic status epilepticus, MDZ—midazolam, PRO—propofol, KTM—ketamine, PTB—pentobarbital, Dex—Dexmedetomidine.

Heterogenous combinations of IVADs were used to maintain burst suppression. Nine patients received monotherapy with propofol alone, twenty-one received propofol with midazolam, three received ketamine in combination with propofol and midazolam, one received pentobarbital with propofol and midazolam, and one received a combination of midazolam and pentobarbital. A summary of the proportion of time during which various combinations of IVADs were administered is shown in (Fig 1b).

Patients also received other medications including blood pressure medications (i.e. antidiretic hormone, labetalol, nicardipine, dopamine and norepinephrine), muscle-relaxants (i.e. cisatracurium and neostigmine), narcotics (i.e. fentanyl) and boluses of anti-epileptic drugs (i.e. lacosamide, fosphenytoin and levetiracetam). These medications were excluded from the analysis because they are not known to influence burst suppression.

### Intra-patient variability in the amount of EEG suppression at constant infusion rates

A total of 108 constant-dose data segments with a median BSP of at least 0.05 were extracted to study intra-patient pharmacokinetic/pharmacodynamic (PK/PD) variability. These segments collectively constituted 897 hours of data. Of these, 54 segments belong to the nRSE group (391 hours) and 54 segments belong to the aRSE group (506 hours). The median duration of the segments is 8.3 hours, the minimum length is 1 hour, and the maximum length is 68.4 hours.

**Fig 2:**
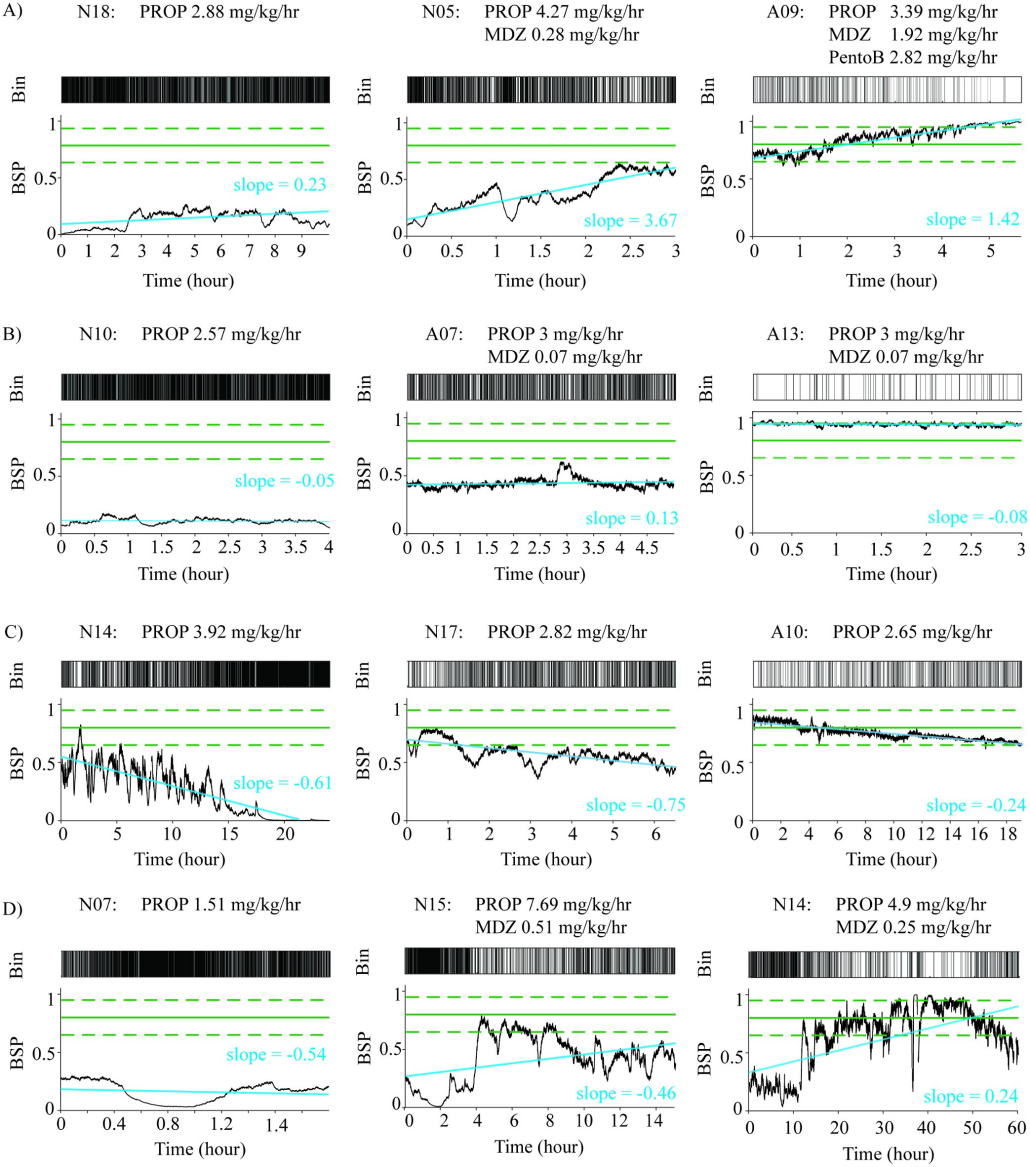
Example BSPs from 12 patients demonstrate the large degree of intra-patient variability in BSP achieved maintained on constant doses of IVADs. Solid green lines indicate the reference of BSP 0.8 and the dashed green lines indicate the reference range 0.65–0.95. The blue line shown is the linear regression line for the BSP data. A) Increasing BSP over time. B) Approximately constant BSP over time. C) Decreasing BSP over time D) Varying up and down over time. For each panel A–D), we deliberately chose examples with low, median and high median BSP values to show that the trends are not unique to BSP ranges.

There was substantial variation in measured BSPs despite constant-IVAD infusion rates. In both aRSE and nRSE patients, we observed four trends: BSP increasing over time, decreasing over time, staying approximately constant over time, and varying over time. The diversity of observed trends is demonstrated in (Fig 2). The slope from the linear fit, which describes the overall change of the measured BSPs in the constant-dose data segments, indicated changed of more than 10% from in an hour in 36.1% of nRSE patients and 10.9% of aRSE patients. Minimum (maximum) observed slopes are -4.25 (6.77) per day for the nRSE group and -4.25 (4.64) per day for the aRSE group. The distributions of slopes observed in the nRSE group and the aRSE group did not differ significantly.

### Between-patient variability in the amount of EEG suppression at constant infusion rates

**Fig 3:**
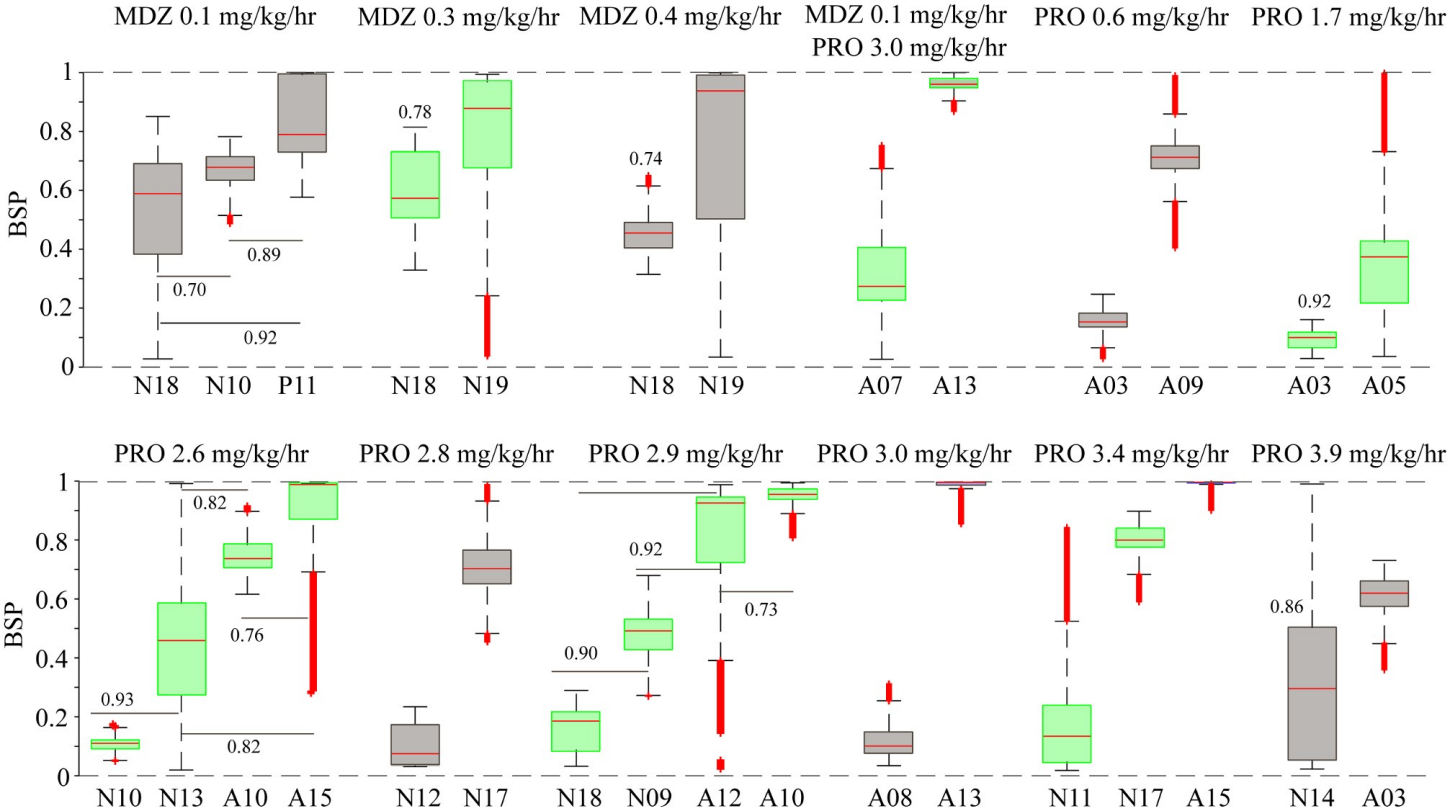
Comparison of BSP values obtained for similar combinations of IVADs given to different patients. We identified twelve groups of patients, who received infusions at the same weight-normalized rates, and compared the distribution of BSPs achieved in patients within each group. Numbers associated with the boxplots indicate the probability of a sample from the distribution with the lower median is smaller than a sample from the distribution with the higher median. Horizontal lines are used to indicate which pairs are being compared for sets with data from more than two patients. Unlabeled pairs within sets are different with probability one. The infusion rates for each patient group were as follows: Patients belonging to groups 1 to 3 received midazolam infusion only at 0.1, 0.3 and 0.4 mg/kg/hr respectively. Patients belonging to group 4 received both midazolam (0.1 mg/kg/hr) and propofol (3.0 mg/kg/hr). Finally, patients belonging to group 5–12 received propofol infusion only at 0.6, 1.7, 2.6, 2.8, 2.9 and 3.0 mg/kg/hr, respectively.

From the constant-dose data segments, we identified 77 distinct weight-normalized dose combinations. Among these, eight pairs, two sets of three, and two sets of four patients received similar dose combinations of IVADs. The measured BSPs for these sets are compared in (Fig 3). The probability that the measured BSPs from members within a set will differ from each other had a range of from 0.7–1 for all comparisons within a set with a median probability of 0.925 (IQR [0.82, 1]). This indicates that it is very probable that different BSP values may result when similar weight-normalized infusion rates of IVAD are given to different patients, i.e., there is substantial inter-patient PK/PD variability.

### Quantitative assessment of the amount of EEG suppressions achieved

**Fig 4:**
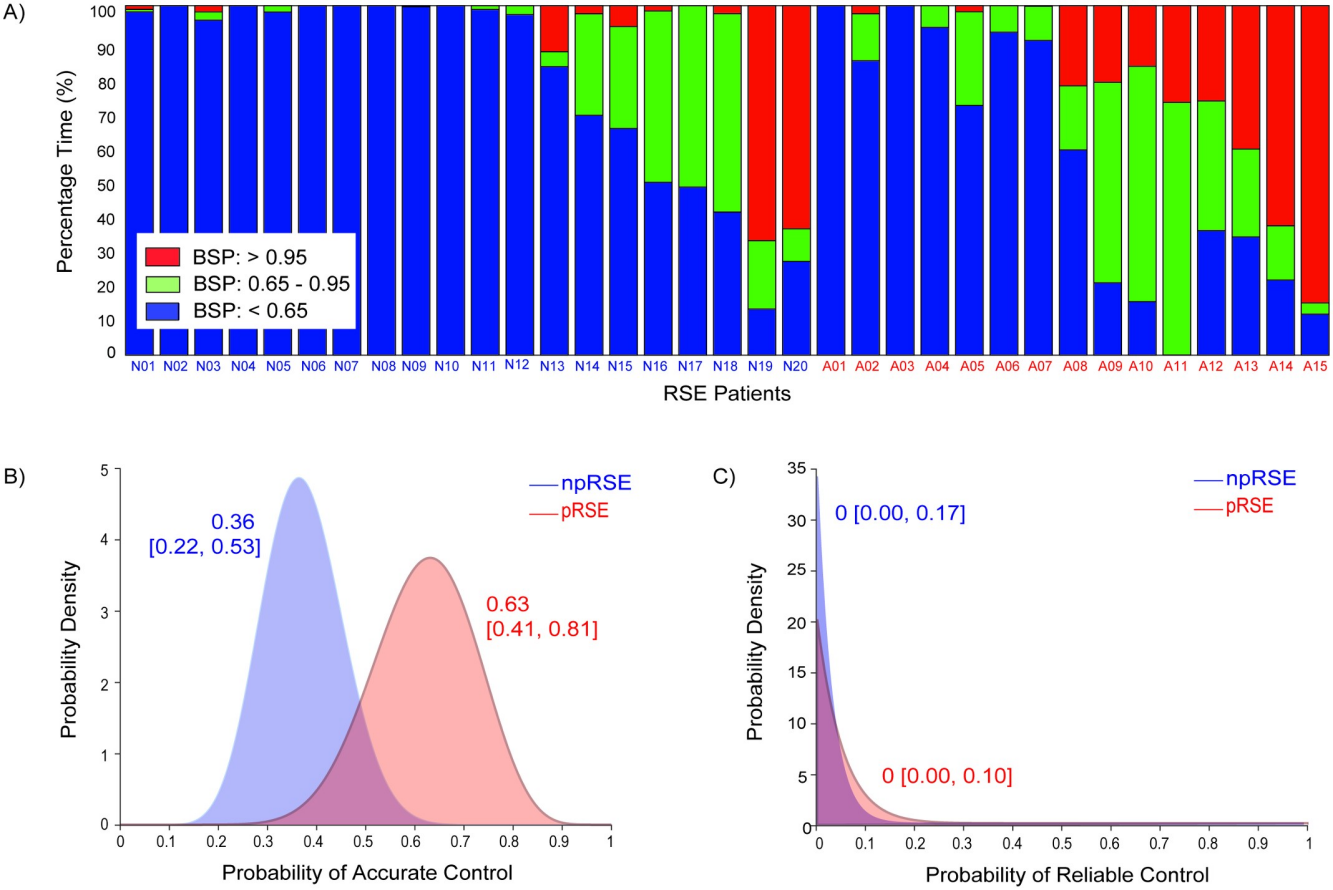
Measures of level of burst suppression achieved. a) Percentage of time spent within and outside of the reference BSP range in individual patients. Indices representing nRSE patients start with ‘N’ and indices representing aRSE start with ‘A’. Ideally, all patients should be on target (green) for 100% of time. Yet in many patients, the measured BSPs fall below the reference range (blue), and in some cases the measured BSPs overshoot the reference range (red). b—c) Posterior probability distributions for the probabilities that BSPs achieved was accurate and reliable in the nRSE and aRSE patient groups. The numbers associated with each distribution are the maximum *a posteriori* estimate and 95% Bayesian credibility intervals of the distributions.

We computed PTa, PTi, and PTb for each patient (see Fig 4a) and found a marked tendency for the measured BSPs to fall below the reference range. Patients remained in the reference range 0–72% of the total time under treatment, with a median PTi of 8% only (Interquartile range, IQR [0, 29] %). Nine of the thirty-five patients never reached the reference range. The median PTa and PTb were 2% (IQR [0, 21] %) and 84% (IQR [37, 100] %) respectively.

The accuracy and reliability of control were also poor in both aRSE and nRSE groups. The clinical goal is to have the probability of accurate and of reliable control to be close to 1. However, as shown in (Fig 4b and 4c), the probabilities of accurate control and of reliable control were 0.632 (95% BCI [0.409, 0.809]) and 0 (95% BCI [0, 0.139]) for the aRSE group, and 0.364 (95% BCI [0.221, 0.534]) and 0 (95% BCI [0, 0.084]) for the nRSE group respectively. The control of BSP was more accurate in the aRSE group than in the nRSE group with a posterior probability of 0.97. We did not find significant difference in the reliability of control between the two patient groups.

## Discussion

Pharmacologically-induced coma is the current standard of care for patients with RSE. Its goal is to induce a state of profound brain inactivation to stop further seizures. Our study quantitatively analyzed the amount of EEG suppression achieved during PIC targeting EEG burst suppression under current management practices using BSP. To assess the variability in the BSP, we used a range of BSPs derived from the institutional clinical guideline as reference. Our results show that the amount of EEG suppression achieved in clinical practice is highly variable. Most commonly, BSPs fell well below the reference range of 0.8 ± 0.15 recommended by the local practice guidelines. In other patients, BSPs persistently remained for prolonged periods above the reference range.

The above results are significant because insufficient attention is given to variability in the amount of EEG suppressions achieved in RSE patients in clinical research. For example, in a randomized single-blind trial, which found no apparent benefit to achieving burst suppression in RSE patients, none of the patients in the burst suppression group were consistently maintained in burst suppression for the full duration of treatment [17]. The study also did not quantify the amount of EEG suppression achieved. In two other retrospective observational studies which suggested that PIC used to treat RSE patients may cause longer hospital stay and higher relative risks for infection, new disability and mortality, patients were divided into groups for comparison depending on whether or not they had received IVADs [18,19], but there was no confirmation of the amount of EEG suppression achieved or their variability.

Our results strongly suggest that outcome studies of RSE patients are confounded by heterogeneity in the level of EEG suppression achieved. If we posit that there exists an optimal level of EEG suppression for RSE management (as the existence of the clinical guideline assumes), falling short of it means insufficient treatment, while exceeding it means that patients are subjected to added risks associated with IVAD exposure without additional therapeutic benefit. Unfortunately, our data do not allow us to evaluate this hypothesis. For example, a reasonable comparison would be to compare outcomes between patients with BSP within the target range, say, >75% of the time, with those with BSP below the target range >75% of the time. However, as can be seen in Fig 2, all patients with aRSE died, regardless of time spent in burst suppression; and in patients with nRSE, no patients were within the target range 75% (or even 50%) of the time. Future studies that control for levels of brain inactivation are necessary to draw valid conclusions on risk-benefit profile of induced-coma.

Many possible reasons may contribute to the observed variability. Despite having an institutional guideline intended to reduce practice variation, clinicians may target a higher or lower BSP depending on other clinical factors such as prior failed attempts to wean a patient off IVAD due to return of seizures or the presence of hypotension. Given a specified target BSP, intermittent patient monitoring with subjective interpretation of EEG and adjustment of infusion rate in a busy ICU setting is also inherently difficult. In addition, our results suggest that the difficulty may arise in part from significant PK/PD variability. We found that similar weight-normalized infusion rates of IVADs resulted in substantially different levels of burst suppression in different patients; the level of burst suppression in patients given constant infusion rates of IVADs can also change substantially over time. Significant PK/PD variability in response to anesthetic drugs is also evident in other studies. One study, for instance, found no significant difference in the mean daily propofol, morphine or fentanyl dose received by critically ill patients who experienced burst suppression vs. patients who did not. [20] These observations indicate that personalized titration of IVADs is crucial for achieving and maintaining a desired amount of brain inactivation.

Furthermore, we also found that the control of a prescribed level of burst suppression was more accurate in aRSE patients than in nRSE patients. aRSE patients often have more diffuse and more severe brain injury compared with nRSE patients [21,22]. Anesthetic drugs might more easily suppress brain activity in a more severely injured, making burst suppression levels “easier” to control. Global anoxic brain injury also damages the metabolic pathways of the brain and reduces the rate of ATP production [23]. This may also increase the propensity for profound brain inactivation by anesthetic drugs [24]. The appropriate amount of IVADs administered to a patient is therefore also a function of disease-specific factors.

The involvement of patient- and disease-specific factors suggest that in order to improve our ability to maintain a consistent level of brain inactivation, we need to tailor the infusion rates of IVADs according to the specific needs and responses of patients at specific times. Our results suggest that infusion rates of IVADs should probably be adjusted more frequently than is currently done. The maximum rate of change of BSP observed in our study was 6.77 per day, or approximately 0.15 every 30 minutes. This suggests a need to adjust the infusion rates of IVADs at least every 30 minutes to keep a patient who starts off on target at a BSP of 0.8 within the target range of 0.65–0.95. In comparison, the median time between adjustments off the infusion rates in our patient cohort is 2.0 hours (IQR [1.0, 4.5] hours).

We did not attempt to determine the patient- and disease- specific factors that can be used to predict IVAD dosing requirements for individuals because this is a limited single center study and the patient population was quite heterogeneous. We did not attempt to determine the optimal amount of EEG suppression for treating RSE patients. As discussed earlier, the high level of intra- patient variability in the degree of EEG suppression achieved would likely confound the result. Therefore, we did not track incidences of adverse events, breakthrough seizures, or patient mortality. These issues are critical to address in future studies. Nevertheless, these limitations do not impact our ability to quantify variability in the levels of brain inactivation achieved in RSE patients.

Finally, while we recommend personalizing dosing and increasing the frequency of IVAD infusion rate adjustment, these ideal practices can be challenging to implement in practice. The existing paradigm for delivering PIC is labor-intensive and skill-dependent. It relies on intermittent manual titration of IVAD infusion rate(s) by ICU staff based on frequent review of the EEG and patient physiological data. Given unpredictable responses to IVADs and the dynamics of the ICU environment, it is improbable that busy ICU staff are able to provide the recommended care event in the best of scenarios. Therefore, we need a new management paradigm to deliver IVAD infusions in a more personalized, precise, and efficient manner, to maintain consistent levels of brain inactivation during PIC.

One promising alternative management paradigm is the use of closed-loop anesthetic delivery systems (CLAD). These systems can continuously monitor the response of patients to IVADs and automatically titrate the infusion rate of IVADs to achieve specific levels of brain inactivation set by physicians. Previous work has shown that CLADs can maintain various levels of burst suppression with substantially higher reliability and accuracy of control compared with the observations made in this study. [10,11,25,26] Our group and other researchers are actively developing ways to improve the safety, usability, and performance of these systems. [26] Quantitative results of variability reported in this study can inform this process and help researchers develop safer systems. These systems may enable future outcome studies of RSE patients that are not confounded by variability in levels of brain inactivation during PIC, and enable us to optimize the treatment.
